# Patient experiences with collaborative practices between primary and specialized care in Norwegian cancer pathways: a qualitative study

**DOI:** 10.1186/s12913-025-13831-1

**Published:** 2025-12-03

**Authors:** Katrin Mork Hove, Anett Skorpen Tarberg, Frøydis Vasset, Mads Solberg

**Affiliations:** 1 Department of Health Sciences, Faculty of Medicine and Health Sciences, NTNU, Ålesund, Norway; 2 Department of Health and Social Sciences, Professor Molde University College, Molde, Norway

**Keywords:** Cancer care, Collaborative practices, Coordination, Patient experiences, Cancer pathways, Integrated care, Qualitative study

## Abstract

**Background:**

Increased complexity and specialization of cancer care and treatments are posing significant challenges for patients navigating different levels and professions in the healthcare system. This shift necessitates coordinated care and collaboration across various healthcare providers. The study explores patient experiences with collaborative practices in clinical cancer pathways (CCPs) and examine their implications for a new initiative in Norwegian cancer care known as Cancer pathway - Home (CPH), which seeks to integrate primary care and core palliative care principles into clinical cancer pathways.

**Methods:**

A qualitative, descriptive design was employed, utilizing semi-structured interviews with 14 patients with cancer who participated in clinical cancer pathways. Our informants were recruited through healthcare professionals and local cancer associations. Data was analyzed using thematic analysis to identify themes and ideal types related to patient perspectives on collaborative practices.

**Findings:**

Participants articulated a necessity for collaboration that addressed both the disease and the patient with the disease. Some participants felt like “packages” in a fragmented system, while others experienced more holistic, patient-centered care, particularly in rehabilitation settings. We propose a typology of four collaborative practices in Norwegian cancer pathways derived from the patient-perspective: ‘fragmented collaboration’, ‘complementary contributions’, ‘team-based collaboration’, and ‘co-navigated, patient-centered collaboration’.

**Conclusion:**

Our patient-derived typology, ranging from ‘fragmented collaboration’ to ‘co-navigated, patient-centered collaboration,’ reveals a clear requirement for more integrated and holistic approaches to cancer care across the continuum of cancer pathways. Patients consistently emphasized the importance of collaboration that addressed both their clinical and psychosocial needs. The Cancer pathway – Home (CPH) initiative aligns with more patient-centered models and shows promise to enhance patient experiences by promoting structured collaboration, improving equity, and coordinating medical and psychosocial support. Addressing persistent challenges in collaboration across primary and specialist care is essential to overcoming fragmentation and realizing the full potential of integrated cancer care.

## Introduction and background

According to the World Health Organization (WHO), cancer poses major public health challenges globally as incidences are rising rapidly [1]. In Norway, the empirical context for this study, over 38,000 cases are diagnosed annually, with an expectation that numbers will exceed 50,000 by 2040. Improvements in early detection and treatments have increased survival rates, where nearly 75% of cancer patients now live five years or longer from diagnosis. Cancer is therefore increasingly viewed as a chronic condition, with many survivors facing a range of biosocial challenges and often complex care needs [2].

In Norway, the universal public healthcare system is tax funded and structured across two levels: specialized care, delivered through hospitals managed by regional health authorities, and primary healthcare, provided by municipalities. Cancer treatments involve complex interventions by various specialists and navigating healthcare services while living with cancer presents significant challenges as health systems become more specialized and intricate [3, 4]. Municipalities are increasingly responsible for providing cancer to a growing patient population, with outpatient treatment increasing, and patients often relying on multiple providers [2].

Consequently, systematic and coordinated collaboration throughout cancer pathways that address transitions between primary and specialized healthcare and often involving multiple health professions, are required to address the risk of fragmented care. These interventions aim to deliver “integrated care” [5], addressing gaps and poor coordination to prevent adverse impacts on patient experiences and outcomes. Effective cancer care necessitates planning, coordination, and allocation of cancer care resources based around patients’ needs. This is vital, since a cancer diagnosis marks a significant turning point, leading to considerable uncertainty [6], with treatment regimens often imposing significant symptoms burdens [7]. In addition, patients with cancer may experience profound impacts on their social lives and identities [8], with potentially deep consequences for their mental well-being [9].

Considerable efforts are therefore being made internationally and in Norway, the empirical setting of this study, to integrate an oncological, “tumor-directed approach” focusing on disease and treatment, with a palliative “host-directed approach” in health services [4, 10, 11]. The goal is more holistic, patient-centered care (PCC). The Lancet Oncology commission, for instance, highlights four key dimensions for integrated cancer care: (1) multifaceted interventions that combine organizational structures with patient-centered plans; (2) interprofessional collaboration and standardized care pathways to manage complex care processes; (3) strong, collaborative relationships among primary, secondary, and tertiary providers; and (4) systematic management of transitions between hospital care and other settings like primary care. Regarding this second dimension, we here adopt the term ‘collaborative practice’ to broadly encompass patient experiences of cross-level and interprofessional care collaborations throughout the cancer pathway. As this focus is central to our study’s aim and research questions, we return to this concept below.

Effective patient-centered care encompasses five key dimensions: consideration of biological, psychological, and social factors; recognition of patients as unique individuals; collaborative decision-making; a strong, trust-based therapeutic alliance; and seamless care across healthcare services [12]. This must build on an understanding of the patient’s experience to foster robust and collaborative provider-patient relationships and effectively coordinating care [3, 12, 13]. Early integration of palliative, patient-centered care into oncological pathways can improve outcomes, including better symptom management and quality of life, facilitating tighter integration in cancer care, regardless of prognosis [14–16].

### Clinical cancer pathways

Clinical cancer pathways (CCPs) are now widespread across many health systems. In Norway, CCPs constitute standardized, nationally regulated protocols for cancer treatment and care, encompassing diagnostics, treatment, follow up and rehabilitation [17]. The National Cancer Strategy 2025–2035 aspires to position Norway as a leader lead in delivering exemplary patient pathways [2]. Between 2015 and 2017, Norway instituted 28 CCPs within its universal public health system to ensure well-organized, comprehensive, and timely care [17].

While the CCPs stress the importance of integrating the patient’s individual needs, qualitative research suggests that patient perspectives and life circumstances are often overlooked, with the focus being on clinical issues surrounding the cancer [18]. While offering predictability, CCPs thus represent a tension between standardization and individualization [6, 19]. Studies by Solberg, Berg, and Andreassen [20–22], for example, highlight challenges for patients and families, who find CCPs fragmented and confusing, especially during transitions that leaves them “lost in the loop” [22], with insufficient support to navigate the pathway’s early stages [20–22].

### Interprofessional collaboration

Interprofessional collaboration (IPC) is essential for integration of health services, as recognized by the Lancet Commission on Palliative Care and Pain Relief [23]. IPC aims to bridge gaps between professional silos across levels, providers, and locations, ensuring coherent service whether the care goal is curative, life-prolonging, symptom relief, or end-of-life care [24]. Collaboration among health professionals is vital to address patient needs, and deliver holistic, integrated healthcare services, crucial for patient-centered cancer care [25–29].

IPC benefits both patients and professionals by fostering collective actions to address complex patient needs and develop mutual respect and trust [30]. Patient-centered practices is the center of IPC, signaling a shift towards integrated care models for complex diseases like cancer, with a potential for both quality improvement and cost rationalization [13, 31–33]. Implementing cancer care coordination strategies may improve care quality and patient outcomes [3]. Here, clearly defined roles, standardized protocols and guidelines, are crucial for overcoming system barriers and enhancing integrated care between primary and hospital settings [33, 34].

Multiple barriers hinder the integration of palliative, holistic approaches and interprofessional collaboration within cancer pathways. These include misconceptions about palliative principles being only for ‘end-of-life’ care, entrenched work practices, inadequate information sharing, resource limitations, and persistent gaps in collaboration between care providers [4, 26, 35, 36].

### The cancer pathway - home initiative

In Norway, a 2018 survey on user-experienced quality, following the implementation of clinical cancer pathways (CCP), revealed shortcomings, highlighting poor communication and coordination between healthcare professionals. A 2022 survey by the Norwegian Cancer Association identified issues like abrupt transitions between specialist and primary care, inadequate needs assessments, and insufficient follow-up.

In 2022, Norwegian municipalities, who provide primary care services, began implementing the Cancer pathway - Home (CPH). Introduced by the Norwegian Directorate of Health [37], in response to criticisms of clinical cancer pathways as overly medicalized and fragmented^1^. This initiative seeks to bridge the gap between specialized cancer treatment provided by hospitals in CCPs, with the responsibilities of municipalities, which provide primary and ongoing supportive cancer care through cancer coordinators, general practitioners and other services. Municipal cancer coordinators in Norway, most often oncology-trained nurses (though occasionally from other health and social care backgrounds), are appointed by municipalities to ensure continuity and coordination of care. They play a key role in supporting patient-centered care by guiding patients through available services, tailoring support to individual needs, maintaining an overview of local resources, and fostering collaboration among healthcare providers, institutions and support services [38]. Championed by stakeholders like the Cancer Society, the expansion aims to integrate primary care services into clinical cancer pathways, that have traditionally centered around specialist care. By incorporating principles of palliative, patient-centered care, the new pathway promotes a more seamless and holistic patient experience across hospital and primary care follow up services [37, 39]. To achieve this, the CPH includes three ‘interaction points’ for assessing patient needs: one immediately after diagnosis (at the hospital), another 3–4 months later (in primary care), and a third 12–18 months later (in primary care), with follow-up as necessary. For instance, CPH incorporates the Distress Thermometer rating scale, to assess and address holistic patient needs, potentially reducing undesirable care variations [11]. This standardized approach aligns with recent European guidelines by ESMO, recommending regular assessment of issues like mental health in cancer care [11].

### Knowledge gaps, aim and research question

Patient experiences and the factors that influence them are increasingly recognized as key concerns for cancer care quality, and is a growing research field [13, 40]. The success of initiatives like CPH hinges on effective interprofessional collaboration (IPC) across levels of care. However, research on how professionals collaborate has largely overlooked the patient perspective [41], with a scarcity of studies from primary care settings [42], including in Norway. To address this knowledge gap, this study was guided by the research question: *How do patients in clinical cancer pathways experience collaborative practices?*

In this study, the term ‘collaborative practice’ is used broadly to encompass patient experiences of cross-level and interprofessional care collaborations throughout the cancer pathway. By addressing this question, the study aims to advance our knowledge of how different collaborative practices shape patients’ care experiences within clinical cancer pathways (CCP) from diagnosis to treatment and follow up phase. Understanding the impact of collaborative practices on patients’ experiences is essential to improving healthcare experiences and outcomes. It also informs efforts towards integration of primary care with patient-centered follow up into clinical cancer pathways, ensuring that resource-intensive initiatives like CPH achieve their intended outcomes.

The article proceeds as follows: first, we present our methods and analysis. Next, we present our empirical findings and identify four ideal-types of collaborative practices. In the discussion, we draw out the implications of these experiences in terms of barriers and opportunities facing the CPH.

## Methods

### Design

We adopted a qualitative, descriptive design with an inductive approach to gather responses grounded in interview data and minimize bias from prior theorization [43]. Our focus was on staying close to the participant’s lived experience, without relying on pre-formed abstract concepts. As with all qualitative research, theme selection is shaped by interpretive choices and researchers’ positionality. The research team brings multidisciplinary and complementary expertise to mitigate potential bias stemming from a single perspective. The first and second authors are certified cancer nurses with extensive clinical experience in oncology care, providing deep contextual insights into primary and specialist cancer care. The third author also has a nursing background and has extensively researched interprofessional collaboration in healthcare and professional education. The last author is an anthropologist and ethnographer with considerable experience in qualitative and interdisciplinary health services research. This diverse research team enhances the study’s credibility and reflexivity to support a balanced interpretation of the data.

Data collection was guided by a semi-structured interview guide. To ensure transparent reporting, the study adheres to Standards for Reporting Qualitative Research guidelines [44].

### Recruitment and setting

Recruitment and data collection were undertaken by the first author between June 2022 and October 2023. Recruitment was a multi-step process (see Table 1), involving collaboration with healthcare professionals in hospital and primary care settings, including local Cancer association in the region of Central Norway.

The first author initiated contact by informing department heads requesting permission and help to distribute information letters to all the cancer departments in the region. This written information was shared with healthcare professionals in three out-patient, hospital-based clinical facilities where patients received treatment. Information was also distributed to primary care professionals, such as cancer coordinators and specialized cancer nursery departments. The first author also provided information about the study in scheduled meetings with health professionals in hospitals, and primary care cancer coordinators.

Healthcare professionals were asked to share written and oral information with eligible patients who were willing and able to share their experiences with the research project. Recruitment letters with information about the study were also distributed to waiting rooms in hospital departments in the three regional hospitals. Additionally, cancer coordinators in primary care services shared information letters at the monthly ‘Cancer Café’, a community event, where patients with cancer gather for social interaction and informal support. This initiative led to the recruitment of six informants (five from the hospitals, and one from primary care).

Further recruitment efforts included outreach to local cancer associations (e.g. for breast, prostate, lung, colon, and hematological/lymphatic cancer). The first author attended meetings in cancer associations to present the study and distribute information about the study to members, recruiting eight participants.

**Table 1:** Recruitment process and sites for recruitment

Recruitment sites	Actions taken	Participants recruited
*Institutional outreach (hospital and primary care)*		*Total: 6*
Hospital departments & polyclinics	Information letters sent by department heads to all cancer departments in the region. Information was shared with health professionals in three local departments and distributed in waiting rooms. The first author presented the study to health professionals.	5 participants (Hospital)
Primary care services	Information shared with local cancer coordinators and special cancer nursery departments.	1 participant (Primary Care)
*Community outreach (cancer associations and events)*		*Total: 8*
Local cancer associations	Phone calls, attending meetings, and distributing information letters. Cancer associations distributed letters to members.	8 participants
Public events	Information letters shared at monthly Cancer Café events.	-
**Total**		**14**

### Participants

Inclusion criteria were patients who currently were, or had previously been, enrolled in clinical cancer pathways, whether in the treatment phase or receiving follow-up care. After receiving information from healthcare professionals and consenting to participate, patients either contacted the first author via telephone or informed the healthcare professional of their interest. The first author then arranged an interview time via SMS or phone call.

The study included 14 participants, four males and nine females, aged 35–70 years. All participants were currently enrolled in or had previously been enrolled in clinical cancer pathways, spanning from diagnosis through treatment, supportive care, and disease-specific rehabilitation. This included patients with both curable disease and those with advanced cancers, some of whom were receiving palliative, life-prolonging treatment. The scope of rehabilitation is broad; in the context of this study, ‘rehabilitation’ was limited to participants’ experiences with physiotherapists in the clinical pathway or with national rehabilitation services. The following pathways were included: breast [4], prostate hematological [1] lymphatic [1], colon [1]. One participant was initially enrolled in a pathway for kidney cancer, and subsequently one for liver cancer. One participant had several cancer diagnoses, being enrolled in pathways that included gynecological, malign melanoma, colon, and breast cancer (see Table 2).

**Table 2:** Overview of informant characteristics. Information about diagnosis, treatment and clinical pathways were based on information provided by the participants

**Age range**	34–70	**Participant ID**	
**Gender distribution**			
Female	9	ID: 1,2,4,6,7,11,12,13,14	
Male	5	ID: 3,5,8,9,10	
**Cancer diagnosis**		**Clinical cancer pathway**	**ID**
Kidney and liver	1 participant	Kidney and liver (surgery)	1
Breast	5 participants	Breast (surgery and chemotherapy and/or radiotherapy and/or hormone therapy)	2,4,6, 12,13, 14
Prostate	3 participants	Prostate (surgery and/ or radiotherapy and/or chemotherapy and/or hormone therapy)	8,9,10
Hematological	1 participant	Hematological (chemotherapy)	11
Lymphatic	1 participant	Lymphatic (chemotherapy)	3
Colon	1 participant	Colon (surgery and chemotherapy)	5
Multiple cancers	1 participant	Multiple gynecological, breast, malign melanoma, colon (surgery)	7

The voluntary nature of CPH and inconsistencies in its rollout meant that patient engagement with municipal cancer services was highly varied. The resulting sample provides a comprehensive snapshot of this landscape, encompassing the full spectrum of patient engagement. The sample included participants formally enrolled in the CPH initiative, others who experienced key components of the model (e.g., cancer coordinators or homecare nurses), and critically, those who had no primary care contact, representing the target population CPH aims to reach. Therefore, rather than being a limitation, the sample composition is a direct reflection of the fragmented state of cancer follow-up during CPH implementation. The varied encounters provide invaluable insights into the broader challenges of care coordination, role ambiguity and information transfer that are central to our analysis and discussion. A more detailed breakdown of participant encounters with health professionals is presented in the findings section.

### Data collection

We collected data during a transitionary period corresponding with the early stages of the nationwide CPH implementation. This timing was crucial, as it allowed us to capture a wide range of patient experiences that reflect the real-world challenges of establishing a new pathway like the CPH.

While not a theory-driven study, the semi-structured interview guide was developed by the first author and refined in collaboration with the last author. It was specifically designed for this study and informed by a rich conceptual and policy landscape. This landscape includes national guidelines for cancer pathways and existing research on patient experiences, particularly concerning collaboration across care levels in cancer care [17, 37, 45]. These resources provided the essential sensitizing concepts that guided the development of interview questions and the subsequent analysis of patient narratives regarding their collaboration experiences within the context of the Clinical Cancer Pathways and the emerging Cancer Pathway Home (CPH) initiative.

The first author conducted and audio-recorded all the interviews. The interview began with open questions about collaborative practices in the cancer pathway and then focused on three key areas of the patient experience: (1) collaboration between hospitals and primary care (between levels of care), (2) collaboration between patients and healthcare professionals (patient-centeredness), and (3) collaboration between different healthcare professions (interprofessional collaboration). The interviewer encouraged the participants to narrate freely to let themes emerge naturally, followed by open-ended questions to explore and ensure coverage of key topics in the interview guide.

Interviews were transcribed locally on a computer using Whisper, a speech recognition system by OpenAI. Transcripts were manually reviewed by the first author, by thoroughly comparing the transcript to the audio-record, to correct errors and ambiguities.

In general, participants found discussing their experiences and sharing their cancer narratives with the interviewer helpful. Many expressed strong sentiments and engagement, attesting to the personal impact of their cancer journey^4^. Following the interviews, six participants reached out to share additional insights, further enriching the data with nuances about their experiences with collaborative experience in cancer care. The interviews varied in length from 30 min to approximately 1 h and 20 min, depending on the participants’ availability, engagement, health condition and capacity.

### Analysis and coding

The analysis adhered to Braun and Clarke’s [46] systematic, six-step principles for thematic analysis. Familiarization with the data: the first author carefully reviewed interview transcripts in detail, cross-checking them against the audio recordings, noting initial ideas and observations. Generation of initial codes: using an inductive approach, the first author engaged in an initial explorative, open coding phase that deeply engaged with the data to ensure analytic rigor. Codes organized using NVIVO software to systematically handle the data. Searching for themes and patterns: data were broken down into meaningful units to identify salient and recurring patterns. Credibility was strengthened through collaborative discussions between the first and last author and detailed review of the initial coding. Reviewing themes and patterns: coding schemes were iteratively refined through more focused coding, and shared between all co-authors for assessment and validation, which informed subsequent revision of the analytical themes. Defining and naming themes: descriptive labels were assigned to text segments in alignment with the empirical data, study aim, and research questions. This iterative process led to the identification of patterns of collaborative practices. The final coding scheme, including translated quotations, subthemes, and themes, was jointly reviewed by the first and last author. Finally, themes and patterns were discussed among all co-authors to ensure accurate representation of patient experiences. To maintain coherence and conceptual clarity, we integrated ideal type construction within the final step of the thematic analysis framework. The four ideal types represent abstractions of the thematic patterns, capturing distinct collaborative dynamics experienced by patients (see Table 3).

**Table 3:** Relationship between core components (themes, subthemes), illustrative quotes and the four ideal types visualized in Fig. 1

Ideal type	Core components (examples of themes, subthemes)	Illustrative quote
Fragmented collaboration -feeling like a package	**Fragmented collaboration and somatic-centered standardization** • *Siloed*,* documentation-driven work*: minimal direct interaction between professionals, relying on written documentation. Reinforces separate roles, silos, and lack of care continuity. • *Patient as sole care coordinator*: patients must repeatedly share their history and manage their own care coordination between different services. • *Standardized procedures over individual needs*: A focus on standardized, somatic tasks overrides patients’ individual needs, leaving emotional and psychosocial concerns unaddressed. • *Poor integration of mental health care*: difficult and delayed access to public mental health services often requiring separate GP referrals	“I don’t think they [health professionals] really cooperate, so it ends up that I don’t think they really cooperate, so in the end you have to tell your story several times” (6) “you’re just a package, the oncologist had a kind of strange attitude, it was just a package, right?” (8)
Complementary contributions	**Independent but tailored contributions** • *Personalized support from general practitioners* highlights the value of a proactive GP who knows the patient’s full history, bridging gaps in the fragmented care pathway. • *Complementary roles across professions*: different professionals work separately but address various aspects of care. While not integrated, their distinct contributions combine to provide tailored support.	“The cancer coordinator has the professional background in relation to the disease and its treatment, while the psychiatric nurse addresses emotional well-being and family concerns. It’s helpful to have these roles separated, as you discuss different topics with each. They complement each other well, and both send updates to the GP and follow up individually. Regular follow-ups with the psychiatric nurse have been very effective, especially during the challenging period after completing cancer treatment. It’s beneficial to have someone to talk to during difficult times as you transition back to everyday life” (14).
Team-based collaboration	**Experiencing team-based collaboration** · *Interprofessional teamwork and continuity* reflect a high degree of integrated collaboration. Work practices are organized around interdisciplinary work, supported by physical co-localization and shared goals. Continuity, availability, and consistent follow-up. · *Dedicated contact person*: a key person plays a central role in identifying needs, supporting the patient, and proactive facilitation of access to interprofessional resources and relational safety. · *Holistic approach*,* beyond illness*: an interprofessional, team-based approach helps address comprehensive, psychosocial and existential challenges of living with cancer. Focus extends beyond the management of the disease.	“There, it’s interdisciplinary with different people in various stages. You are with others in similar situations. You learn about side effects and late effects and get help. They address your individual needs. You get input and feedback and open conversations, they care, follow up, and remember you… only then do you feel seen.” (9)
Co-navigated patient- centered collaboration	**Relational and personalized care** • *Individualized and prepared follow-up*: participants feel acknowledged and supported when specific professionals (often oncologists) know their history and tailors care to the person, not a “package”. • *Proactive and consistent professional engagement*: Value is placed on professionals who reach out beyond medical treatment to check on broader psychosocial needs, creating a sense of safety. • *Hospital-centered trust*: Participants develop strong trust in hospital-based care due to high-quality follow-up, leading them to rely on this setting for their needs.	“I think the most important thing, from a patient perspective, is that we are different. Whether a package or not, we are individual persons, and I feel that I have been allowed to be that” (2) “If I had any need, I would have asked about it at the hospital, because they were unique” (3)

Drawing on the final iteration of recurring patterns and themes identified through our thematic analysis; we selected shared characteristics across the datasets to develop four ideal types of collaborative practices. These types were developed to synthesize the complex collaborative and diverse experiences described by participants, as illustrated in Table 3. This analytical process involved moving from a descriptive analysis of what participants talked about in the interviews, to a more abstract conceptual typology by configuring thematically identified structures that illustrate distinct patterns of interprofessional collaboration in the data. The ideal types were developed across datasets, recognizing that many participants partially fit into more than one type, given their sometimes-varied interprofessional experiences while enrolled in CCP^2^.

**Fig. 1(1.1–1.4):**
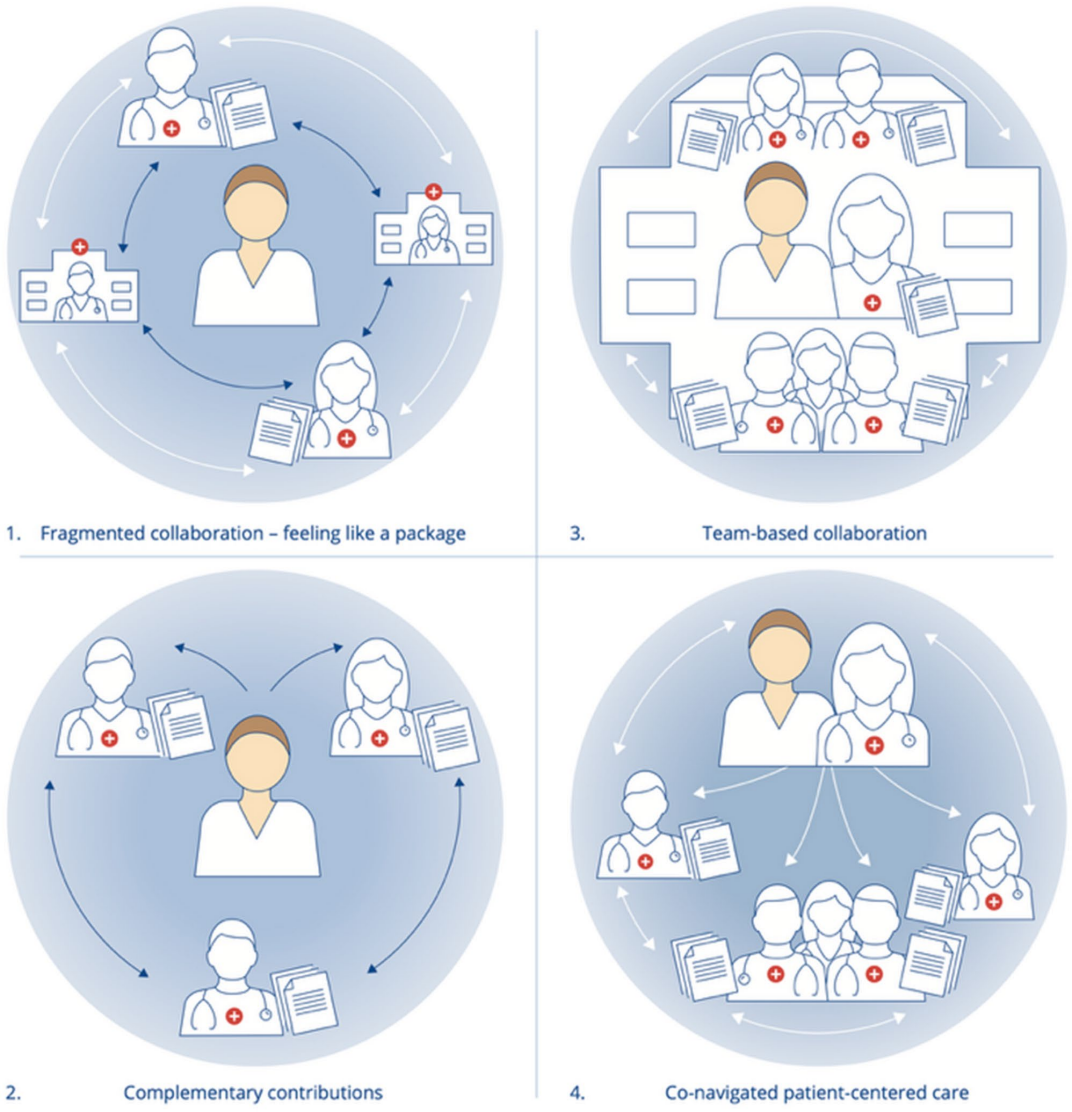
The four ideal typical models of collaborative practice in cancer pathways, based on participants’ accounts

Figure 1; Table 4 visualize and describe these four ideal types in detail.

**Table 4:** Summary of the four ideal types

Ideal type	Description
Fragmented collaboration-feeling like a package	Collaborations occur primarily through referrals and written documentation exchanged among healthcare professionals. Interactions are procedural rather than relational, lacking patient-centered support. Professionals work independently with minimal alignment or coordination. Patients interact with each provider in separate settings and are responsible for coordinating their own care. General practitioners (GPs) typically receive clinical summaries from hospital specialists.
Complementary contributions	Healthcare professionals contribute independently, but their efforts collectively address patient’s needs. The patient engages with healthcare professionals at different levels of the health system, in separate settings and coordination remains patient driven. GPs may receive summaries from various professionals involved in primary care (e.g., cancer coordinators and mental health teams), but direct communication from hospital-based providers is often limited to discharge summaries.
Team-based collaboration	Collaboration occurs within structured, time-limited settings like national rehabilitation centers. Different professionals are co-located, and collaborate actively, reducing the burden on patients to repeat their history. There is focus beyond illness. A dedicated contact person supports the patient, identifies needs and facilitates access to interprofessional team-based resources. This model reflects holistic, patient-centered care and a high degree of interprofessional patient centered coordination.
Co-navigated, patient-centered collaboration	A dedicated professional oversees the patient’s care, treatment and ensures continuous follow-up. This individual facilitates access to various professionals as needed, addressing both medical and psychosocial needs. Care is individualized and coordinated, with patients feeling supported and reassured as professionals co-navigate the pathway alongside them. Collaboration follows a patient-centered approach.

## Findings

In this section, we present findings based on the informant’s descriptions of collaborative practices within clinical cancer pathways.

As mentioned, the voluntary nature of CPH and inconsistencies in its rollout meant that patient engagement with municipal cancer services was highly varied. Table 5 gives a comprehensive snapshot of this landscape, encompassing the full spectrum of patient engagement. Two participants were formally enrolled in the CPH initiative (IDs 6, 14), while others experienced key components of the model, with four having contact with cancer coordinators (IDs 4, 5, 11, 12), and three receiving care through existing municipal structures like homecare nurses (IDs 1, 5, 8). Crucially, the sample also illuminates barriers to CPH uptake by capturing the views of three participants who preferred hospital-based follow-up (IDs 2, 3, 10) and five who had no contact with primary care cancer services at all, representing the very population CPH aims to reach (IDs 1, 7, 8, 9, 13).

**Table 5:** Healthcare institutions and health professions described by participants

Healthcare institution	Health professions
Specialist care/hospitals	Oncologist, Surgeon, Cancer nurse, Physiotherapist, Nutritionist, Radiation therapist, Social worker, various specialists-MDs
Primary care	General practitioner (GP), Cancer nurse, Cancer coordinator, Psychiatric nurse, Home care nurse, Physiotherapist.
National rehabilitation institutions	Interprofessional teams: Social worker, Cancer nurse, Psychologist, Medical doctor, Physiotherapist, Nutritionist
Private healthcare	Psychologist

To illustrate recurring patterns described by participants in our data, we developed four ideal types of collaborative practices. As shown in Fig. 1, ideal type 1 and 2 (left side), reflect situations where participants described themselves as the primary coordinators of their care. In contrast, ideal types 3 and 4 (right side) represent experiences where healthcare professionals assumed a more active coordinating role, often supported by dedicated contact persons and interprofessional resources.

### Fragmented collaboration: feeling like a package (Fig. 1.1)

This ideal type reflects patient experiences where interactions with healthcare professionals, including those providing physiotherapy or respite care within the clinical cancer pathway, felt fragmented and lacked relational collaboration.

Informants described collaborations as primarily relying on written referrals, documentation, and impersonal information exchange. These encounters often addressed clinical treatment, with each professional group mainly contributing individually, based on their expertise (see Table 3). Informants’ examples primarily highlight interactions *within* specialist care or *between* specialist and primary care, seldom detailing the kind of interactive, interdisciplinary teamwork characterized by face-to-face meetings or other forms of integrated coordination. One informant noted how her GP was just copied in on correspondences with cancer specialists via discharge summaries. In her experience, this did not translate into patient-centered interactions: “[I received] nothing through the general practitioner, he has just been listed as a copy recipient here” [6].

Another highlighted the logistical challenges of managing appointments: “in a way you have to be healthy to be sick to keep track of all these appointments” [13]. She vividly described how she tried to navigate the different follow-up consultations, including doctor appointments, treatments at the outpatient clinic, and radiology examinations, while navigating family life:

If you don’t check the mail or keep track of where you’ve put the letter, it can easily slip through. And that’s quite important when you’re undergoing cancer treatment. You might not be as concentrated and focused on the same time [13].

She described using a calendar to track appointments and avoid double-bookings by rescheduling. Weekly treatment led to many inquiries and trips to and from the hospital. Others described reliance on next of kin to keep track of appointments.

Several recounted how they frequently had to interact with unfamiliar healthcare providers throughout the treatment phases. Differing opinions from new doctors often added confusion and uncertainty, making it hard to keep track of treatment plans and information. A recurring issue with fragmented collaborative practices was the necessity of repeatedly recounting personal and medical histories to multiple professionals, which informants found emotionally and practically straining. One informant stated: “I don’t think they [health professionals] really cooperate, so it ends up that I don’t think they really cooperate, so in the end you have to tell your story several times” [5].

Many informants were unaware of collaborative practices within primary cancer care, and which primary care services were available. This lack of clarity made it difficult to see the value of adding more services to an already demanding cancer pathway. Informant 2 expressed this hesitance as follows:

It seems unclear what municipal cancer care should contribute to the cancer pathways. I had very little contact with the municipality. Actually, I haven’t had any contact with my home municipality at all. I know that there is a cancer coordinator.

For others, initiating contact with the cancer coordinator appeared inherently challenging: “Nurses in the cancer department at the outpatient clinic said that there was an offer in the municipality with a cancer nurse. So, I got a phone number where they said I could call her. I felt it was very difficult to take the initiative to contact her myself” [3].

Informant 6 reflected on a visit from the cancer coordinator, who assessed her needs using a questionnaire tool, with results indicating extensive mental health needs, including depression:

I wish she had said, ‘I see you score so high, you check yes, you cry a lot, you check yes for being depressed, you score high on the mental health scale, indicating you are not doing well. We need to do something to get you some help. We, in the municipality, when it is a Cancer pathway - Home, need to follow up. We need to find some tools you can use in your daily life to improve your well-being. Should we bring in a psychologist’? [5].

She then questioned the support services, asking: “What’s the point of having cancer coordinators in the municipalities if they don’t even have a list of names they can refer cancer patients to?” [5].

Concerns about the adequacy of pathway support services also extended to other professional fields. For instance, some informants experienced challenges with physiotherapy, a well-established service in the cancer pathway. Some found physiotherapy too demanding, citing the intensity of the cancer treatment, and the emotional strain of being around other cancer patients. Informant 13, for example, recalled the training “was too much” and how she needed “to get distracted” because being “in a room with many others without hair” made her “focus on the fact that I was sick.” Instead, she wished that physiotherapy services were made available after completing cancer treatment.

Participants generally expected care providers to focus primarily on somatic issues, a mindset that often led to their emotional and psychosocial concerns being overlooked and was rarely challenged. This expectation was reflected in their experiences; for instance, collaborations with cancer nurses typically centered on clinical procedures, treatment advice, and guidance related to cancer diagnoses and side effects, rather than broader psychosocial support. Consequently, many informants voiced a critical need for psychologists to be integrated into cancer pathways to address mental health struggles, advocating for more interdisciplinary resources, particularly mental health support, within municipal cancer care.

Further underscoring this unmet need, several informants described a lack of interaction with healthcare professionals equipped to address the mental and emotional burdens of their cancer diagnosis and treatment. They often attributed this perceived fragmentation in their care, where emotional support was largely absent, to healthcare professionals lacking the capacity, the appropriate perspective, or sufficient time for the kind of patient-centered interactions that would provide such support.

All informants described the emotional toll of a cancer diagnosis as traumatic, overwhelming, chaotic, marked by survival uncertainty, fear of progression and recurrence. Cancer treatments were physically demanding, exacerbating mental distress and significantly impacting their quality of life. This emotional upheaval disrupted daily lives and relationships, reshaping roles and identities, leaving them feeling vulnerable. Informant 5 called it “a dramatic total package”, describing profound changes in roles as a grandfather, partner, brother, son, and worker. Informant 9 described it as “the world came crashing down,” with “no guarantees about the future”. Informant 4 framed the emotional turmoil around anxiety: “You get that fear of death and feel all the things and become afraid of everything. I think it is connected to the cancer diagnosis… that part is also very important, that no one caught that. I find that very, very strange.”

This profound emotional impact was compounded by a perceived lack of collaborative practices, which made the cancer pathway more demanding than necessary. Several informants highlighted the absence of someone to talk to, no offers of assistance from the outpatient clinic, or access to a psychologist. This unmet need for emotional support from the start, coupled with fragmented collaborative practices, left many feeling isolated and forced to cope with the mental strain on their own. Although cancer pathways are intended to be patient-centered, several informants noted how their journey primarily focused on disease and treatment. This emphasis shaped collaboration practices with healthcare professionals, leaving their psychosocial support needs unmet. Informant 9 bluntly remarked, “the cancer department only sees the disease; it’s only when you’re half-dead that you get noticed”. Similarly, Informant 8 described the care as impersonal: “you’re just a package, the oncologist had a kind of strange attitude, it was just a package, right?“. Informant 14 added: “it kind of feels like you come in, get treatment, and then you just go home. They don’t ask about anything else”. These statements reflect a perceived lack of patient-centered collaboration, with care focused on the illness not recognizing the individual behind the diagnosis. Post-treatment, informants also reported enduring physical side effects such as neuropathy, leaks, and fatigue, besides mental health issues, realizing that life would never be the same. Informant 6 described this as carrying “an extra brick in your backpack,” reflecting a constant fear of relapse. She desired “tools to process grief and trauma”, noting how patients are not always able to think clearly and handle distress on their own.

While informants acknowledged effective referrals from oncologists for the physical aspects of cancer care (e.g. physiotherapists and nutritionists), access to mental health services was notably lacking. There appeared to be a significant gap in collaboration between oncologists and mental health professionals. Many identified psychologists as crucial for emotional and psychosocial support, yet these were largely unavailable in the public health system, forcing patients to rely on often unaffordable private options. This issue was especially pronounced for patients with pre-existing mental health challenges, who noted that the cancer diagnosis intensified existing health issues such as anxiety and trauma. In their view, these issues were not adequately addressed and collaboratively managed by the health professionals in their pathway. Informants described how this lack of interprofessional practice worsened their psychological burdens and impaired their coping abilities in a vulnerable situation. Notably, they described how oncologists would refer them back to GPs instead of directly to psychiatric specialists, requiring separate GP appointments for referrals. This led to significant delays, as patients often had to wait both for GP consultations and for mental health care, leaving many feeling that their psychological distress was neglected until it reached a critical point.

### Complementary contributions (Fig. 1.2)

In contrast to fragmented collaboration practices, some informants described an ideal type with more individually tailored interactions. In this model of collaborative practice, different professionals contributed independently but effectively to cancer care.

For instance, some noted how physiotherapists had played a key role in recovery from cancer-related injuries and pain management. Informant 8, for instance, noted how his physiotherapist got him “on my feet in the end”, starting with brief sessions lying on the floor, progressing to joint training sessions as part of his cancer recovery. Others noted the complimentary contributions from social workers in Cancer pathway - Home. Informant 14 shared how referrals to a social worker at the cancer department gave crucial support, and how things “went well with regards to the sick leave, NAV [the labour and welfare administration] and the job”, as well as childcare and financial concerns. Social workers were described as particularly effective collaborators in addressing practical challenges, offering advice on financial matters, patient rights and obligations, thereby easing these burdens in cancer care.

For some, General Practitioners (GPs) provided crucial resources for coping with the cancer pathway, providing significant support for patients’ psychosocial needs and burdens, addressing gaps in emotional and psychosocial care left unfulfilled by other health professionals. They expressed valuing the GPs familiarity with their personal circumstances, and their emphatic understanding of the stress associated with cancer. Some respondents described how their GP even made unannounced calls to check on their well-being, a highly valued gesture. Informant 4 described how the GP “picked me up”, making her “function at work and get rid of the [negative] thoughts”. She emphasized how the GP “has been absolutely crucial… that she [GP] followed me up the way she did. God, if she had not been there, what would have happened, in a way”. The strong, long-standing relationship with her GP played a crucial therapeutic role in managing her mental health when other professionals were not available. Informant 10 noted: “My GP deserves praise; he keeps track, he follows me up properly”. This sentiment was echoed by Informant 3: “I know my GP… He was on vacation when I got the diagnosis, so he has only read everything in the medical records… So, he sent a message and asked if I could drop by so we could talk about it, and I said yes”. Similarly, Informant 5 valued proactive support from the GP: “He actually calls from time to time, regardless of whether I get in touch or not, and asks how things are going. I think that was alright”.

These examples show how a proactive, personalized approach supported the informants during a vulnerable and demanding phase of their cancer pathway. This could also come from other professions. Informant 14, for example, shared an experience of having follow-up appointments with both a psychiatric nurse and a cancer nurse, noting their complementary functions:

The cancer coordinator has the professional background in relation to the disease and its treatment, while the psychiatric nurse addresses emotional well-being and family concerns. It’s helpful to have these roles separated, as you discuss different topics with each. They complement each other well, and both send updates to the GP and follow up individually. Regular follow-ups with the psychiatric nurse have been very effective, especially during the challenging period after completing cancer treatment. It’s beneficial to have someone to talk to during difficult times as you transition back to everyday life.

These experiences emphasized the importance of personalized, role-specific contributions. Although health professionals operated in distinct roles, without much interprofessional collaboration, they addressed the patients’ evolving needs in a complementary manner throughout the cancer pathway. Together these efforts enabled personalized and supportive healthcare. In their examples, informants described how emotional support and personalized care were tailored to specific needs. Working separately, but complementary, the professionals ensured comprehensive support throughout the cancer pathway.

### Team-based collaboration (Fig. 1.3)

A recurring and striking theme across our informants’ narratives was that national rehabilitation facilities were *the only settings* described as providing truly holistic, interprofessional and patient-centered collaboration. None of our informants reported any experience with municipal rehabilitation services. National rehabilitation services, while not part of the standardized pathways, are services that patients can request access to via GP-referrals, usually after the treatment stage. A sharp contrast was drawn between the experiences of holistic, interprofessional collaborations in rehabilitation institutions, and the more fragmented engagements encountered in the clinical cancer pathway. In the interviews, rehabilitation centers were associated with continuity, availability, and cultivating strong relationships with health professionals, which are characteristics of effective, patient-centered collaborative practices. Interprofessional collaboration in these centers helped address the comprehensive psychosocial challenges of living with cancer. Informant 9 contrasted collaborative practices in CCPs with the rehabilitation experience:

There, it’s interdisciplinary with different people in various stages. You are with others in similar situations. You learn about side effects and late effects and get help. They address your individual needs. You get input and feedback and open conversations; they care, follow up, and remember you… only then do you feel seen. At rehabilitation, something happens! They do not work with the illness but with everything else.

One informant reflected on the value of being recognized as an individual by a dedicated professional:

…the great thing about rehabilitation is that you get a contact person who is, how should I say, your primary person. You have conversations about how you feel your handling life, a conversation partner. A bit about your challenges and such things… It was so safe, because they, I didn’t need to use words for it. They see it in you… [10]

Both these accounts reflect how a team-based interprofessional collaboration, centered on the whole person, cultivated a sense of being seen and understood, supporting the many dimensions of living with cancer.

### Co-navigated, patient-centered collaboration (Fig. 1.4)

The last ideal type centers on experiences where our informants felt acknowledged, supported, and well-cared for within the clinical cancer pathway. It reflects a model of collaboration characterized by relational and individualized care, with a proactive engagement from designated professionals, and care continuity. These experiences contrasted with standardized and fragmented collaboration patterns that participants found less supportive. This was attributed to healthcare professionals adopting a patient-centered, collaborative approach that valued individualized attention over rigid standardization. Notable examples involved how oncologists in the clinical cancer pathway proactively addressing not just medical treatment of disease, but also psychosocial concerns. By being familiar with their personal and medical history and addressing their broader needs, these professionals made patients feel truly seen and heard in the CCP. Informant 2 reflected on this as follows: “They [health professionals] need to find out who they have in front of them… spend time figuring out who it is and try different approaches… it will be unique from person to person”. This perspective contrasted with the feeling of being treated as a generic “package”, a perception elaborated by Informant 2: “I think the most important thing, from a patient perspective, is that we are different. Whether a package or not, we are individual persons, and I feel that I have been allowed to be that”. Informant 1 echoed this sentiment: “The doctor sees me as a human being, not like being A4”, invoking a powerful metaphor contrasting personalized care with the impersonal nature of highly standardized printing paper.

A recurring subtheme highlighted by the participants was the value of predictability from professionals both initiating contact and providing consistent follow-up. Informant 6 illustrated this, noting her oncologist: “called several times during my chemotherapy to check on how I was doing, and when I was feeling as bad as I was, she really followed up with me, I must say… she sees me”. Similarly, other informants found stability and support when professionals were prepared and maintained continuity of care by consistently inquiring about their well-being and progress.

Knowing a patient’s history, actively listening, providing tailored information, and addressing unique needs were described by our informants as key features of positive relational interactions. They found reassurance in knowing that help and guidance were available when needed.

Notably, these positive collaborative practices were primarily described within hospital settings and achieved through consistent professionals who coordinated care across services in the hospital, when needed. In the words of Informant 3: “If I had any need, I would have asked about it at the hospital, because they were unique”. Notably, informants sharing these experiences did not voice a demand for *additional* municipal health and care services, instead relying on hospital professionals, whom they found reliable, accessible and adequately patient-centered.

## Discussion

Our data explores patients’ perspectives on how health professionals work together across professional boundaries and levels of care in cancer pathways, and how these practices affect their care. The findings reveal considerable variation in how patients experience collaborative practices. While some informants described positive, individualized interactions with specific professionals, many perceived the pathways as lacking adequate interprofessional collaboration and patient-centered care. Communications between healthcare providers often occurred indirectly, relying on written referrals and documentation, rather than in-person teamwork. As illustrated in Fig. 1.1*Fragmented collaboration*; *feeling like a package*, patients interacted between profession-specific silos, with little integration across professional roles. Despite expectations that professionals with diverse competencies would collaborate to meet their needs, informants struggled to articulate what such collaboration should involve, particularly who should coordinate care, and which professionals should be engaged. This uncertainty often left patients managing their own care, repeatedly explaining their needs to different providers and navigating a fragmented referral system with minimal support. These siloed practices further diminished opportunities for meaningful conversations during treatment. This resonates with recent qualitative research from Norway that highlights the tension between standardization and individuation in cancer pathways, often overlooking holistic care needs [6, 22].

A discernable gap identified in our qualitative data was the lack of mental health support, which informants regarded as essential for managing the psychological impact of cancer. Many reported how their emotional struggles were overlooked and attributed this to fragmented collaboration across care providers. These observations confirm previous research indicative that cancer diagnoses often mark a critical life juncture, characterized by uncertainty [6, 18, 22], combined with substantial symptom burdens during treatment [7]. Although depression and anxiety among cancer patients are prevalent, they often go unaddressed, negatively affecting treatment adherence, recovery and quality of life [18]. Our findings reflect these challenges, revealing gaps in mental health support within standardized yet fragmented cancer pathways. Many patients also experienced a predominant focus on biomedical treatment, at the expense of patient-centered care. Fragmentation across professional silos further exuberated these challenges, resulting in lack of integrated care with insufficient attention to the emotional and mental dimensions of their cancer. Notably, experiences of fragmented collaboration reinforced participants’ perceptions of rigid professional roles, leading them to expect care providers to address only specific, and primarily somatic aspects, neglecting more holistic concerns. These expectations were rarely challenged by the healthcare system.

Lacking systematic support, these patients navigated a complex, opaque health system, compounding their challenges. These findings resonate with those of Gorin et al. [3], who highlight the difficulties patients face in navigating complex healthcare systems. They also echo Solberg et al. [22], who found that patients often feel ‘lost in the loop’ amid fragmented and poorly coordinated care transitions as fragmented and confusing. Delilovic et al. [36] documented how weak collaboration and coordination can hinder seamless cancer care. Similarly, studies have shown that communication between GPs and specialists is often disrupted by disorganized referrals and missing key information, such as treatment intent and available alternatives [47, 48]. Resonating with our informant’s experiences, this study highlights the need for more structured and tailored communication practices, as timely and complete information exchange appears essential for continuity across care settings. Informants described persistent fragmentation with professionals often focused narrowly on their specializations, overlooking the broader context of patient needs. This led to repeated recounting of medical histories, inconsistent or conflicting information, requiring patients to interpret isolated bits of information and support, resulting in incohesive care plans [32].

These experiences contrast with instances where professionals effectively complemented each other’s skills to meet patients’ needs. As illustrated in *Complementary contributions* (Fig. 1.2), these patients were often left to act as central navigators of their own cancer pathway yet perceived interdependent professional interactions as genuinely collaborative and responsive in addressing their needs. These examples underscore the inconsistent nature of referrals and written communication. While some patients benefited from complementary professional roles, others experienced fragmented care, leading them to feel like “packages” instead of individuals. These findings align with a recent systematic review by Alessy et al. [13], demonstrating that both individual and structural factors influence cancer patients’ care experiences, with positive patient-provider relationships linked to better outcomes. Differing experiences may reflect how professionals interacted, either engaging in patient-centered collaborative care, or adhering to task-specific routines.

Tensions between standardized and individualized care in terms of collaborative practice were evident in varying patient experiences. For instance, physiotherapy ranged from standardized interventions some informants found overwhelming, to personalized approaches experienced as supportive. GPs who addressed psychosocial needs also exemplify this variation. Similar variation has been found in lung cancer care, where patients who primarily identified oncology teams as their main providers, expressed a clear desire for GP involvement in care coordination and as emotional support [49]. These examples underscore a tension between the predictability offered by standardized care and the need for individualized patient-centered interaction, which remains a persistent challenge for both integrated cancer care [4], and interprofessional collaboration [24].

The third ideal type identified in our material reflects a more integrated approach to care. Unlike the fragmented or complementary models, this type reflects patients experiences with dedicated health professionals, who were knowledgeable about their specific cancer pathway, seamlessly integrating additional expertise as needed. Several informants praised the team-based ‘rehabilitation model’ (1.3), as an exemplary instance with structured, time-limited patient-centered, interprofessional collaboration. Informants emphasized the accessibility and expertise of interprofessional teams, which reduced the need to repeatedly recount their histories to new providers. This sharply contrasted with the fragmented collaboration experience often reported within the clinical cancer pathway (CCP). Recent ASCO guidelines (2024) underscore the importance of interprofessional teams in advancing holistic care, while Alessy et al. [13] highlight how system organization, resource availability, respectful treatment, and patient centeredness shape patient experiences. Informants who accessed integrated rehabilitation models reinforced the value of structured interprofessional collaboration in addressing both medical and psychosocial needs. However, as these models were confined to resource-intensive rehabilitation settings, their broader applicability for improving quality and reducing costs in cancer pathways remains uncertain [31], despite the strong interprofessional element noted. Interprofessional collaboration is a cornerstone of palliative care, supporting the delivery of patient-centered, holistic care. When applied in oncology, such approaches have been linked to improved outcomes and life quality [14–16]. Notably, our data suggests that elements of this collaborative, holistic approach aligned with palliative values, could also emerge in mono-professional consultation, particularly with oncologists.

In the fourth ideal type, *Co-navigated*,* patient-centered collab*oration, informants described healthcare professionals as adopting a holistic, individualized approach, that fostered confidence in accessing additional support when needed. Reflecting palliative care principles, this model addressed both the disease and the person. Informants described these collaborations as providing stability and predictability amid the uncertainties of diagnosis, treatment, and follow-up. Similar to type 3, it featured proactive, consistent engagement, accurate record keeping, and meaningful dialogue that addressed emotional and psychosocial concerns, reflecting the importance of strong interpersonal skills among healthcare professionals for truly patient-centered interactions [50].

Notably, these collaborative practices were mainly described in hospital settings, and interactions with oncologists particularly, who were viewed as the most reliable, accessible, and patient-centered providers. However, uniquely reliance on hospital services may also lead to gaps in post-treatment support. A Swedish study [51], for example, highlights how access to primary care in municipalities often depends on patients’ individual capacities and initiative. This underscores a need for improved coordination between specialist and primary care. Strengthening links between hospitals and community-based services can promote structured, equitable support and reduce overdependence on hospital follow-up. Co-navigation of cancer pathways should therefore actively integrate primary care resources to support patients. Effective communication and engagement are known to be vital components in integrated cancer care [33]. The co-navigated collaborative practice described here aligns with patient-centered care principles (PCC) [3] and may overcome the persistent challenge of care fragmentation [3, 24]. Moreover, PCC enhances care quality, patient self-efficacy, and promotes trust in cancer-related information [52]. Additionally, coordination interventions that support patient navigation have been found to nearly double the likelihood of effective healthcare utilization, reducing delays, improving health outcomes, and fostering more supportive care environments [3].

The patient experiences around collaborative practices in clinical cancer pathways embodied by the four ideal types described here reinforce the urgency of addressing continuity gaps in the cancer service [53]. Ideal type one (*Fragmented collaboration*) and two (*Complementary contributions*) often lack dimensions of patient-centered, holistic care, treating care processes as a series of disconnected events that patients must navigate and coordinate independently. In contrast, the third (*Team-based collaboration*), and fourth (*Co-navigated*,* patient-centered collaboration*) reflect more integrated models, consistent with both IPC principles [25–27, 29] and integrated care [28, 53]. In these models, patients reported support from dedicated professional’s familiar with their cancer pathway, who worked collaboratively to integrate additional competencies as needed. Professionals actively co-navigate the cancer pathway alongside the patient. The third illustrates team-based services where multiple competencies are coordinated around individual cases in a time-limited setting, while the fourth, depicts a model where healthcare professionals jointly ensure comprehensive follow-up, maintain care oversight, and seek additional expertise in collaboration with the patient. From the informant’s perspective, patient-centered continuity and co-navigation facilitated positive collaborative experiences.

These findings underscore how both individual and structural factors shape patients’ experiences with collaborative practice. Our study demonstrates how the enactment of fragmented, complementary, or co-navigated, patient-centered collaborative practices in daily clinical interactions shapes patient experiences. Likely, these dynamics, as embodied by our ideal types, are also consequential for patient outcomes.

### Challenges and prospects for CPH

Our findings provide valuable insights into barriers and opportunities facing the Cancer pathway - Home (CPH) initiative, which emphasizes patient-centered care from diagnosis onward and structured interprofessional collaboration, including an expanded role of primary care. While previous studies have highlighted key factors influencing implementation and offered practice and policy recommendations [34], our findings extend this work. Exploring how interprofessional collaboration shape patient experiences offer new insights into how policy interventions like CPH can reduce fragmentation between primary and specialist care.

The variety of informants experiences with primary cancer care provide critical insights into collaboration challenges that persist across care settings, particularly regarding the role of municipal cancer care support and services.

Fragmented collaboration between hospitals and primary care professionals was a recurring issue, with patients reporting insufficient information and guidance about municipal cancer services, especially during the critical early stages of their cancer pathway. These challenges are not unique to the CPH context; rather, they represent fundamental barriers that need addressing to ensure integration and sustainability of structured care pathways such as CPH.

Many informants were unaware about primary care options beyond their GP, and those who were informed faced barriers, such as high thresholds for initiating contact with cancer coordinators. Others preferred hospital follow-up. Consequently, many patients missed out on beneficial primary care resources. This reflects “role ambiguity” [54] — a well-documented challenge for healthcare organizations [55] where unclear expectations, boundaries, or responsibilities creates uncertainty. The ambiguities revolved around unclear or poorly defined roles of municipal services. This led to uncertainty about the availability, purpose, and integration of municipal services in their cancer pathways, leading to disengagement, causing patients to prefer and rely on hospital-based services. Such ambiguity may undermine the potential for complementary care coordination, leaving patients to navigate complex needs across fragmented services [49, 51]. Clearer delineation of roles, like that between primary care providers and oncologists, could help reduce confusion and ensure care coordination [33, 34].

When combined with role ambiguity, the first two ideal types can significantly detract from patient experiences within the CPH initiative. ASCO guidelines emphasize the importance of early palliative care discussions by oncologists to challenge outdated perceptions [10]. Effective information sharing and communication between primary and secondary care are vital to support for people living with and beyond cancer [33]. The CPH initiative would benefit from early, hospital-led communication about available municipal cancer services, promoting more seamless integration of patient-centered care from diagnosis [10]. Structured follow-ups within the Cancer pathway - Home, can help patients better understand and access these services during diagnosis, treatment and follow-up phases.

Systematic needs assessment during the three scheduled CPH meetings also presents an opportunity to address the full spectrum of cancer-related challenges described by our informants, including psychosocial, emotional, and work or school challenges, from diagnosis onward. Standardized protocols and guidelines for follow-up are necessary to ensure consistency and quality of care [33, 34]. Use of structured Patient-Centered Care (PCC) tools can reduce reliance on individual professional discretion, ensuring more consistent and equitable care. Regular follow-ups support comprehensive care planning, continuity, and may facilitate smoother transitions between hospital and primary care. Still, our data suggests a need for closer collaboration between hospitals and primary care to ensure co-navigated, patient-centered support.

Variability in primary cancer care follow-up underscores the potential of the CPH to improve care transitions and strengthen the care continuum. However, the findings indicate that formalized pathways alone are insufficient to enhance collaborative patient experiences. Addressing the many persistent barriers to effective collaborative practice highlighted in our findings is essential. Accordingly, the success of the CPH initiative hinges on strengthening interdependence through robust inter-organizational coordination and clear communication routines to ensure equitable resource access across levels of care to achieve truly patient-centered outcomes.

The ideal types of collaborative practices developed in this study offer a novel lens for understanding the complex interprofessional and cross-level interactions of collaboration in cancer pathway and how they link to diverse patient experiences. By distinguishing patterns of fragmentation and integration, the ideal types clarify where collaborative efforts fail to integrate and where they align with the patient centered holistic care from the patient experience. This could help pinpoint critical areas where integration is most needed and guide targeted integration within pathways. While the first and second ideal type ([1]– [2]) exemplify fragmentation and barriers to collaboration, the third and fourth ([3]) reflects key principles as outlined by The Lancet Commission [4], and the spirit of the CPH initiative, pointing towards more integrated care models.

## Limitations

This study has several limitations. First, our data collection was confined to a single health region, concentrating on patients in the diagnosis, treatment, and follow-up phases of the cancer pathway, and their experiences with collaborative practices. This may limit the transferability of the findings to other regions or healthcare systems with different organizational structures and resources.

Additionally, the initial coding was conducted by a single author. While formal independent dual coding was not performed, rigor and analytical credibility were ensured through a systematic process of multi-author validation. This involved joint assessment and validation of the final coding scheme by the first and last author, and collective reflection and discussion of emergent codes and themes by all co-authors throughout the analytical process.

Our sample may be subject to self-selection bias, as those with particularly strong or reflective experiences may have been more motivated to participate. This could influence the range and tone of perspectives captured.

Further, the study exclusively reflects the patient perspective. While this is essential for understanding user experiences, it does not include the viewpoints of health professionals and caregivers, which is critical for a comprehensive understanding of collaborative practices. These perspectives are being explored in ongoing research by the authors.

The findings reflect a specific period during the implementation of clinical cancer pathways (CPH) in Norway. As these pathways continue to evolve, future studies may reveal different patterns of collaboration and patient experience.

Finally, qualitative research is inherently interpretive. While we employed rigorous methods to ensure credibility and trustworthiness, including reflexivity and peer debriefing, the findings are shaped by the context and the researcher-participant interaction.

## Conclusion

Based on qualitative interviews with patients enrolled in clinical cancer pathways in Norway, this study contributes to the growing body of research on integrated cancer care by highlighting how collaborative practices shape patient experiences. From the patient perspective, collaboration throughout the clinical cancer pathway should be grounded in the core principles of palliative care, being interdisciplinary, holistic and patient-centered, addressing physical, emotional, psychosocial and spiritual needs, alongside disease-directed management. The findings showed that referrals to mental health care were more complex and less accessible than other services, despite its recognized importance in cancer care.

We identified four ideal types of collaboration experiences, revealing significant variation influenced not only by structural barriers such as fragmented, silo-based care and unclear communication, but also by individual healthcare providers approaches to collaboration. The presence of a co-navigator emerged as a key support factor, across these models. Early communication, structured follow-ups, and partnerships with cancer coordinators, GPs, and primary care services, including mental health support, appear to foster more integrated and responsive interprofessional collaboration in cancer pathways.

These findings may inform efforts to strengthen the implementation of the Cancer pathway - Home (CPH) initiative and improve transitions between hospital-based care and primary care services. While some aspects of interprofessional collaboration can be addressed through structural reforms, attention must also be given to how collaborative practice unfolds in everyday clinical settings. Building on this, our findings offer actionable insights for strengthening interdependences between hospitals and primary care that can inform and support ongoing efforts like CPH to achieve more coordinated, and patient centered care. While undoubtedly relevant, this study has not explored the impact of variations between municipalities. It is plausible that the distribution of resources and expertise, including factors such as size and geography, may shape collaborative practices; this, however, remains an issue to be explored in future work.

The ideal types developed in this study offer a conceptual tool for understanding key dimensions of patient experiences with collaborative practices and may support ongoing efforts to achieve more integrated and holistic cancer care.

## Data Availability

The datasets generated and/or analyzed during the current study are not publicly available due to privacy and ethical restrictions but are available from the corresponding author in anonymous form, on reasonable request.

## Footnotes

1 The Directorate of Health received the task of developing Cancer pathway - Home from the Ministry of Health and Social Care in March 2018. The proposal was sent to a “public hearing”, to solicit feedback on the proposal in September 2021. A revised national guideline was published December 29th, 2022.

2 While sharing the conceptual aim of developing a typology, i.e. categories to organize cases or patterns based on shared features, our methodology differs from Ideal-Type Analysis as developed by Stapley et a. (2022). In contrast to ITA, which focuses on identifying distinct groupings of participants based on whole, comprehensive accounts, our thematic analysis looked for patterns across participants’ experiences, focused on more specific elements and themes).

## Abbreviations

CCP: Clinical Cancer Pathways
CPH: Cancer Pathway-Home
IPC: Interprofessional Collaboration
PCC: Patient-centered care
GP: General Practitioner
